# Effects of Medicinal Fungi-Derived β-Glucan on Tumor Progression

**DOI:** 10.3390/jof7040250

**Published:** 2021-03-25

**Authors:** Vaclav Vetvicka, Tamara V. Teplyakova, Alexandra B. Shintyapina, Tatiana A. Korolenko

**Affiliations:** 1 Department of Pathology, University of Louisville, Louisville, KY 630117, USA; 2 State Research Center of Virology and Biotechnology VECTOR, Koltsovo, 630559 Novosibirsk, Russia; teplyakova@vector.nsc.ru; 3 Federal Research Center of Fundamental and Translational Medicine, Federal State Budget Scientific Institution, 630117 Novosibirsk, Russia; shintyapina@yandex.ru; 4 Laboratory of Experimental Models of Neurodegeneration, Scientific Research Institute of Neurosciences and Medicine, Federal State Budgetary Scientific Institution, 4 Timakov St., 630117 Novosibirsk, Russia; t.a.korolenko@physiol.ru

**Keywords:** β-glucan, mushrooms, cancer, immune, health

## Abstract

β-Glucans have been studied in animal species, from earthworms to humans. They form a heterogenous group of glucose polymers found in fungi, plants, bacteria, and seaweed. β-Glucans have slowly emerged as an important target for the recognition of pathogens. In the current review, we highlight the major roles of mushroom-derived β-glucans on cancer progression.

## 1. Introduction

To use natural products as a possible remedy is weaved into the history of mankind. People have been appealing to nature to cure various diseases since ancient times. The first documented history of plant preparation and medicinal use is a record on Sumerian clay tablets from the period of 4000 BCE. There is also an approximately 5000-year-old written Indian document about medicinal effects of mushrooms. Indian Ayurveda and traditional Chinese medicine can serve as examples of healing trends which have been developed through empirical experience. A physician of Marcus Aurelius emphatically pioneered the treatment of diseases by using the specific diets of Galen of Pergamum. Several centuries later, J. Lind [1] used 200-year-old Dutch knowledge of the benefits of citrus fruits to the health of the sailors on long voyages, and conducted one of the first large-scale clinical medical trials. Japanese legend indicates that monkeys without cancer or any other disease fed on the mushroom *Lentinula edodes.* Medicinal plants are commonly used for therapy of various diseases and represent a significant part of old folk remedies. The healing properties of mushrooms have been known for hundreds of years. The number of mushroom types is estimated to be 140,000—of which only about 10% are known.

**Figure 1:**
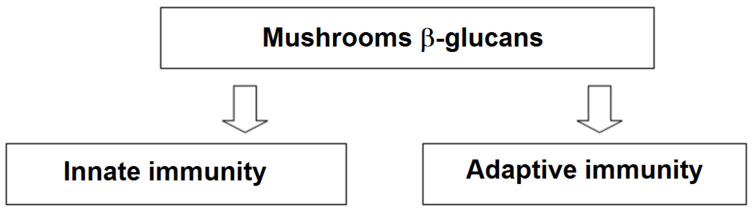
Mushroom-derived β-glucans affect all branches of the immune system.

In the last 40 years, there has been an ever-increasing interest in the evaluation and use of natural products to reduce the risk of numerous diseases or to treat them directly. Bioactive polysaccharides, often isolated from various mushrooms, functioning as biological response modifiers, have quickly become the most studied natural immunomodulator. β-Glucans are glucose polymers present in the cell wall of yeast, fungi, and mushrooms [2]. β-Glucans possess broad immunomodulatory properties, including activation of innate immune functions such as the oxidative burst and adaptive immunity (Figure 1) [3,4,5]. Research on zymosan beginning in the 1940s was followed by the investigation of β-glucans in the 1960s and 1970s, when scientists established the significant influence of β-glucans on the immune system in relation to cancer treatment, anti-infection immunity, restoration of damaged bone, and the activation of innate-immunity cells (macrophages, dendritic cells, granulocytes, and natural killer (NK) cells) [6]. This activation triggers responses of adaptive-immunity cells, such as CD4^+^ or CD8^+^ T cells and B cells, resulting in the inhibition of tumor growth and metastasis [7]. Nonetheless, information on the mechanism of antimetastatic action of β-glucans remains limited.

**Figure 2:**
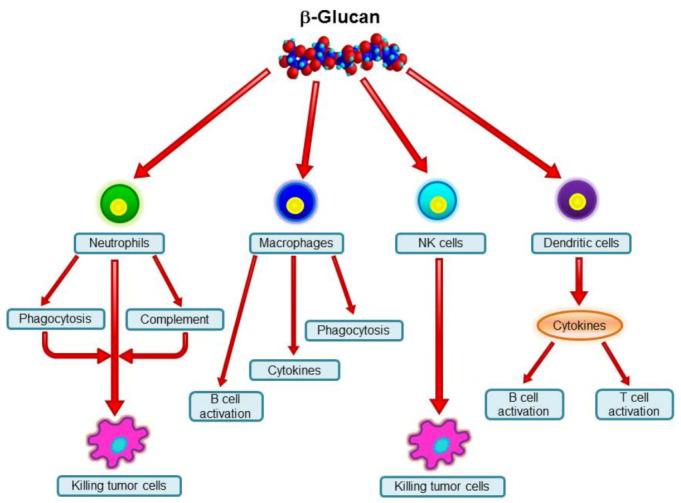
Major effects of β-glucans on immune cells.

A variety of polysaccharides from an array of sources can stimulate the immune system. β-Glucans are among these biological response modifiers, and their biological activities are well established (for review, see Vetvicka 2013 [8]). Some confusion is found among the more than 30,000 research papers, which is most probably caused by differences in source, primary structure, branching, and molecular weight [9,10,11]. The most pronounced effects were found in cancer [7,12] and infection [13] models, but improvements in wound healing [14], inflammation [15], stress [16], immunotoxicity [17], genoprotection [18], viral infection [19], and allergy [20,21] were also found. For a review of the various biological effects of β-glucan, see Vannucci et al. (2013) [22]. The role of the various cell types is summarized in Figure 2.

**Figure 3:**
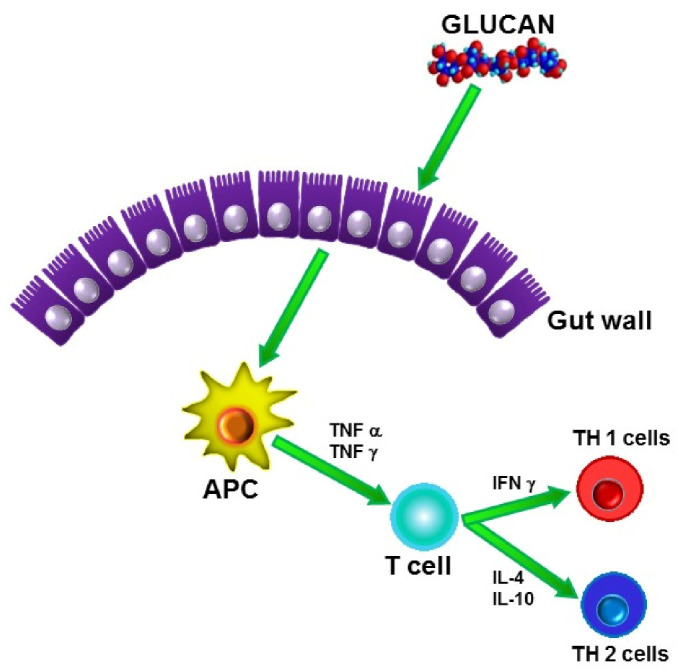
Transport of β-glucan through gut wall.

In addition, β-glucans have been found to be active in every species tested, in earthworms [23], bees [24], shrimps [25], fish [26], chicken [27], mice, rats [28], rabbits, guinea pigs [29], sheep, pigs [30], cattle [31], and humans. These wide-ranging activities make β-glucan probably the only natural immunomodulator able to activate immune reactions across all species. The fact that β-glucans can easily penetrate the gut wall helps their recognition by other immunocytes and subsequent spreading throughout the body (Figure 3).

The fungal kingdom is significant. It not only provides food directly to humans but has also been a source of important drugs; mushroom compounds show promise in cancer immunotherapy [32]. β-Glucans are a group of polysaccharides belonging to the class known as biological response modifiers. They have potential therapeutic use for atherosclerosis, inflammatory disease, type 2 diabetes mellitus, and cancer [33,34,35]. Some hold promise as a new tool for vaccine development [36]. β-Glucans can serve as cellular structural polysaccharides or polysaccharides secreted on the surface of cells (in mushrooms or fungi) [37].

There are many types and sources of β-glucans; some have been studied more thoroughly as possible therapeutic compounds, but many need further research. β-(1→3)-Glucans trigger different immune responses, and these polysaccharides have been shown to be effective immunostimulatory agents [38,39,40,41,42]. The mechanism behind the beneficial effect of β-glucans and other macrophage stimulators administered via different routes has not yet been clearly identified.

Mushrooms have been used in health care for treating simple and common diseases, like skin diseases and pandemic diseases such as AIDS. There are many investigated beneficial effects of β-glucans, including antioxidant, anti-inflammatory, anti-hypercholesterolemic, anti-viral and anti-cancer [43]. They are rich in carbohydrates, like β- and α-glucans, chitin, hemicellulose, mannans, xylans, and galactans, which make them the right choice for prebiotics. Not all medicinal effects of mushrooms, particularly the effects in cancer treatment, can be precisely explained by the action of one single molecule [32].

Hypercholesterolemia is a major risk factor of atherosclerosis and cardiovascular diseases [44]. Because statin use can be associated with muscle problems and other adverse effects, nonadherence and discontinuation of statin therapy are common and often lead to inadequate control of plasma cholesterol levels, increasing cardiovascular risk. In such situations, the hypocholesterolemic effects of β-glucans and other polysaccharides are attractive.

Various hypolipidemic agents are currently in use, including proprotein convertase subtilisin/kexin type 9 (PCSK9) inhibitors, apolipoprotein B-100 antisense oligonucleotides, cholesteryl ester transfer protein (CETP) inhibitors, and microsomal triglyceride transfer protein (MTTP) inhibitors, as well as yeast polysaccharides (β-glucans and mannans) and compounds derived from natural sources (nutraceuticals), such as glucomannans, plant sterols, berberine, and red yeast rice.

In diabetes mellitus, increased β-glucan intake correlates with improved glycemic control, which is associated with slower progression of this disease. There are differences in the effects on glycemic control and insulin sensitivity between oat (whole and bran) extract and β-glucan extract (taken orally) [45].

In cancer, dietary β-glucans can be used as soluble fiber with potential health-promoting effects. These compounds are believed to act on gut peptides, which are important signaling molecules in the regulation of energy and glucose homeostasis [46]. Fungotherapy for targeted treatment of cancer without harmful effects on healthy tissues is actively being developed, utilizing the active ingredients and mechanisms of action of mushrooms [47]. For the subsequent creation of drugs, the need for further studies on the mechanisms of antitumor/antiviral action of the components of medicinal mushrooms is great—for those growing under natural conditions and for those cultivated by solid-phase and submerged methods under laboratory and factory conditions [48].

## 2. β-Glucans Isolated from Different Sources

During 50 decades of research, countless types of β-glucan have been isolated, characterized, and tested. In the scientific literature, you can find several hundred different biological components all under the name β-glucan. There are numerous sources for glucan, from yeast to mushrooms to grain. β-Glucans can be relatively easily isolated from yeast, and the popularity of *Saccharomyces cerevisiae* is based on its availability and low cost. The main raison d’être of β-glucan is to form integral parts of the cell wall. Different physicochemical parameters (solubility, primary structure, molecular weight, branching, polymer charge) play a role in determining whether the polysaccharide modulates immune reactions. Some conclusions can be made. Branched or linear 1,4-β-glucans have very limited activity, if any. β-Glucans with 1,6 configurations usually have limited activity. The highest stimulation of defense reactions has been achieved with β-glucans that have a 1,3 configuration with additional branching at the position 0–6 of the 1,3-linked d-glucose residues. Among all β-glucans, those with a 1,3 configuration are best characterized in the literature. Excellent reviews of the relationship between structure and functional activity have been published [9,49]. For detailed insight into the chemistry, structure, and synthesis of β-glucans, see William et al. (2013) [50]. However, it is important to be mindful that no conclusive agreement on which composition or structure results in the most active glucan has yet been reached. Most likely because the precise structural confirmation of β-glucan in solution, usually due to free and independent rotation of glycosidic bonds between individual residues, is unknown. From the computer models we can assume a left- or right-handed triple helix configuration in water [42]. In fungal glucans, some chemical modifications, such as carboxymethylation, result in improvements of biological activity, particularly in antioxidant activity [51].

The choice of source is more historical than science-based. Investigation of β-glucans began in the 60s of the last century. Two lines can be traced in β-glucans history, based on different starting points, but slowly converging. The first one took place mainly in the USA and Europe, the second one in Asia, specifically Japan. Research on β-glucans in the Euro-American milieu was based on knowledge of immunomodulatory effects of zymosan [52]. When zymosan was examined in detail, β-glucan was identified as a primary effective component. It was subsequently isolated, and the immunological effects were investigated [50,53]. In Asian medicine, consuming different medicinal mushrooms has a long tradition. Initial investigations conducted by Goro Chihara, who isolated lentinan from the shiitake mushroom (*Lentinus edodes*, now *Lentinula edodes* [54]), have led to approval of β-glucan as an official drug. For a summary of mushroom β-glucan action as immunomodulators of cancer immunotherapy, see Ayeka (2018) [32].

### 2.1. Differences in β-Glucan Based on Its Sources

The major structural feature of mushroom β-glucans is a β-1,3-d-glucan main chain with standalone d-glucosyl residues that are β-1,3-linked along this main chain [55]. Some of this glucan can be extracted from the fruiting body of the mushroom, and soluble β-glucans are also produced by cultured mycelia [56]. These medicinal mushrooms are either commercially farmed or collected from the wild. In addition to the traditional production of fruiting bodies, a small percentage of mushroom-derived β-glucans is obtained from mycelia extract produced by submerged fermentation. Properties of these β-glucans depend not only on the species of mushroom or type of isolation, but also on structure, molecular weight, and most of all, purity.

Because β-glucans are not synthesized in the human body, they are recognized as foreign by the immune system and induce both adaptive and innate immune responses [57]. In this context, the digestibility and bioactivity of mushroom extracts with soluble β-glucans, as opposed to the consumption of the whole fruiting body, has been studied and reported [58,59]. In addition to β-glucans, chitin and α-glucans are present in mushrooms; total polysaccharide content of mushrooms ranges between 50% and 90% [60]. The determination of exact β-glucan content is still difficult because an optimal method is still lacking.

Recent studies have revealed that European traditional medical mushrooms have a promising and unique pharmacological potential mediated by known mechanisms (antitumor, anti-inflammatory, antioxidative, and antibacterial effects) [61]. Investigators have discussed the possible usefulness of researching the mushrooms growing in European countries; the number of recent chemical, biological, and pharmacological studies is relatively small, and some mushroom species have not been studied at all. There are certain traditional uses of plants and medicinal mushrooms in Russia; this knowledge can possibly be applied in future studies aimed at safe, evidence-based application of traditional Russian medicinal plants and mushrooms in European and global phyto- and fungo-pharmacotherapy, as well as for the discovery of lead compounds for drug development [62].

### 2.2. Mushroom β-Glucans

Among antitumor treatments, special attention has been paid to mushroom polysaccharides [63,64]. The review article by Wasser (2017) [64] contains a table of therapeutic activity extracts and compounds from medicinal mushrooms, including polysaccharides, evaluated in clinical trials. The data on mushrooms’ numerous bioactive polysaccharides and/or polysaccharide-protein complexes, described from medicinal mushrooms, appear to enhance innate and cell-mediated immune responses, and exhibit antitumor activities in animals and humans. Particularly, and most important for modern medicine, are polysaccharides and low-molecular-weight secondary metabolites with antitumor and immunostimulating properties.

Mushrooms with distinctive fruiting bodies and with significant cancer-fighting abilities belong (with few exceptions) to the class of Basidiomycetes, with the first study published more than 60 years ago [65]. They include *Tremella fuciformis, Grifola frondosa, Lentinus edodes, Agaricus blazei, Ganoderma lucidum,* and several others (for review, see Meng et al. (2016) [66]). Natural modulators isolated from over 30 different mushroom species have shown significant anti-cancer activity [67], but only a limited number progress to clinical trials. Many mushroom-based β-glucans names are based on the name of the mushroom species; the most studied β-glucans are summarized in Table 1.

Schizophyllan (β1-3, 1-6-d-glucan) with a molecular weight of 4.3 × 10^6^ Da, isolated from *Schizophyllum commune,* was found to lower the number of metastases and to improve the lifespan in a Lewis lung cancer model, probably via higher infiltration of T lymphocytes and macrophages [68]. This β-glucan has lately been used on treatment of advanced cervical cancer, where it improves the function of T helper lymphocytes and enhances the IL-2/IL-2R system [69].

Grifolan is isolated from *Grifola frondosa* and usually exists as a linear triple-helical structure. Effects of this β-glucan on cancer development have been studied since 1985, starting with the evaluation of inhibition of sarcoma growth [70]. The summary of subsequent studies using both animal and human models is reported by Hetland et al. (2020) [71]. Most of these studies concluded that this β-glucan stimulates the immune system via an increase of NK cell activity, stimulation of T helper 1 (Th1) response, and suppression of T helper 2 (Th2) response [72]. The most studied version is the so-called D-fraction, which might be either 1-6, 1-4-d-glucan or the more common β1-3, 1-6-d-glucan. The name “D-fraction” came from the process where this fraction was prepared from the crude hot water extract by deproteination. In clinical trials, grifolan caused significant improvements or even regression in 68% of breast cancer patients and 62% of lung cancer patients [73].

Maitake β-glucan is also isolated from the same mushroom species as grifolan. Well-documented anti-cancer effects are supported by enhanced granulopoiesis, increased production of G-CSF, and modulation of CXCR4/SDF-1 expression [74]. Even as these probably have no direct effect on cancer suppression, they represent a valuable tool for modulating the negative side effects caused by chemotherapy and irradiation.

Another important mushroom-derived β-glucan is isolated from *Agaricus blazei.* This glucan was found to inhibit pulmonary and peritoneal metastasis [75]. However, its effects in anticancer immunity have been studied for a much longer period, starting with fibrosarcoma in a double-grafted murine model [76]. The preclinical effects of glucan from *Agaricus* on numerous animal models were summarized by Hetland, Tangen, Mahmood, Mirlashari, Nissen-Meyer, Nentwich, Therkelsen, Tjonnfjord, and Johnson (2020) [71]. Its immunomodulating effects were simultaneously tested in several clinical trials, mostly with solid, albeit not spectacular, effects [77,78].

Additional β-glucan is isolated from cultures of *Ganoderma lucidum*. Using a Lewis lung carcinoma model, oral supplementation with this β-glucan increased NK cell activity, resulting in inhibition of primary tumor metastasis. In addition, it protected the animals against side effects of regular treatment [79]. Long-term supplementation resulted in inhibition of sarcoma growth, probably via changes in immune reaction, particularly by restoration of a balance between cellular and humoral immunity [80]. A recent detailed study of the modulatory activities found anti-inflammatory and immunomodulatory effects manifested via activation of NK cells, macrophages, neutrophils, and dendritic cells [81].

PSP is glucan-based polysaccharide-peptide isolated from *Coriolus* (*Polyporus*) *versicolor*. It has been routinely used in clinical practice in Japan since 1977, and in China since 1987 [82]. It is approximately 62% polysaccharide and 38% protein. A summary of over 40 studies found that stimulation of lymphocytes and macrophages increased production of several cytokines such as TNF-α, IL-1β, and IL-6; significantly increased infiltration of tumors with T lymphocytes and dendritic cells; and improved the side effects of chemotherapy [83]. This β-glucan has been repeatedly evaluated in clinical trials, usually in stomach and colorectal cancer patients, with significant improvements of survival and quality of life (for review, see Kidd 2000 [84]). Isolated Krestin (also known as PSK) is also from the same species. Its composition is 75% β-glucan and 25% protein. Krestin is now used in human patients, where it improves the treatment via stimulation of NK cell activity [85]. A systematic review of its use in lung cancer patients found reduction of symptoms, improvements of immune reaction, and extended survival. Similar results were also found in cases of breast, stomach, nasopharynx, colon, rectum, and esophageal cancer patients [86].

Significant attention is also focused on glucan obtained from *Pleurotus ostreatus.* Recent studies have revealed significant stimulation of NK cell activity oriented towards cancer cells. Detailed analysis suggested involvement of modulation of NKG2D, KIR2DL, and cytokine secretion [87]. This glucan was also found to have antiproliferative and pro-apoptotic effects on cancer cells [88].

Lentinan is isolated from *Lentinula edodes* (also known as shiitake), and its antitumor activities have been known since the 1960s. Later studies revealed that it is a β1,3, 1,6-d-glucan with unusually high molecular weight, reaching 1000 kDa. It is a β-glucan having two β1,6 glucopyranoside branches for every five β(1,3)-glucose residues [89]. Upon original purification, lentinan was found to support cytolytic activity of macrophages towards several types of cancer cells [90]. Immunomodulatory properties of lentinan are well-established and include activation of dendritic cells and macrophages, increase of cytotoxic activity of NK cells, and changes of the Th1-Th2 balance [91,92]. Lentinan was found to increase the production of TNF-α, IL-1β, IL-10, and IL-12 (for review, see Zaidman et al. (2005) [93]). Detailed studies have revealed that the possible mechanisms of lentinan are manifested via activation of immunocytes through several signaling pathways, including Syk-PKC, NF-κB, and TLR4-MAPK. In China, is it routinely used in the treatment of lung, gastric, colorectal, ovarian, pancreatic, nasopharyngeal, duodenal, and cervical cancers [94].

In clinical practice, lentinan is used mostly for gastric cancer, whereas, in its experimental stage, it is being evaluated in a wide range of cancer types, including sarcoma, colon, lung, liver, bladder, and pancreas cancer. In gastric cancer, simultaneous treatment with lentinan and a chemotherapeutic drug resulted in significant improvement of anticancer effects [95]. The addition of lentinan produced favorable effects, such as improvement in the quality of life, increased survival, and reduction of side effects, in almost all studies. One interesting study found that the effects of lentinan were the best in patients with a lentinan-binding monocyte value over 2% [96]. Similar results were found in patients with colorectal cancer [97]. In lung cancer patients, strong improvement in both native and specific immunity, lower side effects, and improvement in the quality of life were reported [98]. A comprehensive review of the last 12 years of literature on lentinan and cancer therapy was summarized by Vannucci et al. (2017) [99]. The authors concluded that the effects of lentinan include stimulation of cytotoxic effects of various cell types, induction of apoptosis by regulation of inflammasomes and telomerase, and change of the Th1 response to Th2 response. Clinical studies showed increased survival strongly correlated with the binding to monocytes. In addition, oral administration was as good as parenteral routes.

Several mushroom compounds have proceeded through phase IV clinical trials and are used extensively and successfully in Asia to treat various cancers and other diseases. Approximately 300 clinical studies have been conducted on *G. lucidum* and some other species of the genus *Ganoderma*. The largest number of clinical trials were performed mainly using *G. lucidum, L. edodes*, *G. frondosa, T. versicolor*, *Phellinus linteus*, and *Agaricus brasiliensis* [64,100]. In many cases, mushrooms were used as adjuvant treatment with conventional chemo- or radiotherapy for various cancers; there was a correlation between their antitumor and antiviral activities.

## 3. Mechanisms of Action

Modern chemotherapy of various tumors by cytostatic agents has shown solid efficacy against most cancers; however, further improvement of this approach is limited by adverse effects of antitumor therapy, namely by low selectivity of antitumor drugs, which is related to their toxicity (adverse effects and complications). In this regard, β-glucan has many biological activities and functions such as stimulation of the immune system and anti-inflammatory, antimicrobial, anti-infective, antiviral, antitumor, antioxidant, anticoagulant, cholesterol-lowering, radioprotective, and wound-healing properties [94,101]. This approach, especially chemically modified water-soluble polysaccharides in combination with antitumor drugs, seems appealing for further use in clinical oncology.

**Figure 4:**
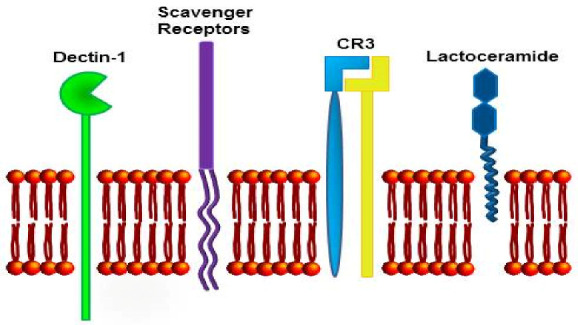
Major β-glucans-binding receptors.

β-Glucans are recognized by numerous membrane receptors (Figure 4), which often share common characteristics. The most important β-glucan receptors are Dectin-1, CR3 receptor, and Toll-like receptors. Dectin-1 receptors react to the β-glucan binding by phosphorylation of the tyrosine-based activating motif [102]. The signaling events involve activation of Syk and NF-κB pathways [103]. For detailed information of molecular interactions of β-glucan with Dectin-1, see Legentil, Paris, Ballet, Trouvelot, Daire, Vetvicka, and Ferrieres (2015) [42].

Another important β-glucan receptor is CR3, known also as Mac-1, α_M_ β_2_-integrin, or CD11b/CD18. This receptor is a one-membrane glycoprotein made up of two noncovalently linked α and β subunits known as CD11b or α_M_ and CD18 or β_2_. With respect to β-glucan binding, the key finding was that β-glucan bound to the lectin domain of CR3, and it primed the receptor in response to tumors that bore iC3b and were normally resistant to cellular cytotoxicity. Many human tumors generate an immune response that results in the deposition of antitumor antibodies, leading to the discovery of the significant therapeutic efficacy of combining β-glucan with antitumor monoclonal antibodies. This significant synergy has been demonstrated in a variety of murine tumors [104], as well as in human carcinoma xenograft models [105,106]. Another experimental proof of concept was reached when β-glucan-mediated therapeutic efficacy was restored by passive immunization with either natural antibodies in SCID mice or antitumor monoclonal antibodies in mice with low titers of natural antitumor antibodies. For more details on the mechanisms of effects on cancer suppression, see Li et al. (2010) [107]. However, the full explanation of how β-glucan binds to its receptor/s, and which receptor is more important for its biological effect, is still missing.

Another mechanism is the effect on myeloid-derived suppressor cells. β-glucan was found to stimulate the apoptosis of polymorphonuclear macrophages and dendritic cells and to regulate the differentiation of monocytic macrophages and dendritic cells [108].

An interesting suggestion was presented recently by Geller et al. (2019) [109]. These authors investigated the complex mechanisms at play within the tumor microenvironment and emphasized the need for the development of strategies that target immune cells within the tumor microenvironment. One such intervention can be β-glucan, a natural compound with an immunostimulatory and immunomodulatory potential and therapeutic anticancer effects. β-glucan can modulate the tumor microenvironment both by bridging innate and adaptive immunity and by switching the phenotype of immunosuppressive cells to an immunostimulatory one. A new role for β-glucan in cancer therapy has been suggested because of an evolving understanding that β-glucan participates in a phenomenon called trained immunity, where innate-immunity cells take on memory phenotypes. This new concept suggests that β-glucan may play an essential part in the prevention and suppression of the growth of various tumors; these effects are important in cancer therapy [109].

Anti-inflammatory activities of some medicinal mushrooms in gut inflammation were shown by Ishimoto et al. (2018) [110]. Those authors performed autodigestion of *Ganoderma lingzhi* and found an enhanced release of hypotensive peptides and an immunomodulatory β-1,3-glucan. Gut inflammation was assessed by measuring the lengths of the intestines and colon, and sepsis was evaluated by means of the survival of the animals.

A main feature of β-glucans is their capacity to function as biological response modifiers, exerting regulatory effects on inflammation and shaping the effector functions of different innate and adaptive immunity cell populations. The potential to interfere with processes involved in the development or control of cancer makes β-glucans interesting candidates as adjuvants in antitumor therapies, as well as in cancer prevention strategies [111]. The recognition by innate immunity cells occurs via ligation of specific PRR, such as Toll-like and C-type lectin-like receptors. Among the latter, Dectin-1 is the best characterized receptor, reported to bind β-glucan from different sources, and is expressed on the surface of monocytes, macrophages, neutrophils, and DC and T lymphocytes [111]. Other receptors, including lactosylceramide receptor, mannose receptor, and complement and scavenger receptors were reported to directly bind β-glucan or to cooperate with Dectin-1 for its recognition; β-glucan was shown to stimulate NK cell cytotoxic activity through direct binding to the NKp30 activating receptor. In vitro, β-glucans can enhance the functional activity of monocytes/macrophages and DC, and activate antimicrobial activity of mononuclear cells and neutrophils.

Mushroom β-glucan may immunomodulate the tumor-associated macrophages in such tumors, like Lewis lung carcinoma [6]. Authors have shown the efficacious effect of mushroom polysaccharides for ameliorating the immune suppression in the tumor microenvironment. Increased M1 phenotype of tumor-associated macrophages and attenuated M2 phenotype of tumor-associated macrophages could be achieved by ingesting mushroom polysaccharides.

The structure of *Grifola fondosa* polysaccharide was identified to be a β-d-(1,3)-linked glucan backbone with a single β-d-(1,6)-linked glucopyranosyl residue branched at C-6 on every third residue. This polysaccharide could interact with poly(A) moiety of a designed antisense oligonucleotide targeting the primary transcript of proinflammatory cytokine TNFα (TNFα-A60). This *Grifola fondosa* polysaccharide-based complex could incorporate TNFα-A60 into the macrophage cells via Dectin-1 receptor and attenuate lipopolysaccharide-induced secretion of TNFα. It was concluded that GFPS could be applied to deliver therapeutic oligonucleotides for the treatment of diseases such as inflammation and cancers [112]. So, β-glucan from *Grifola frondosa* effectively delivers therapeutic oligonucleotide into cells via Dectin-1 receptor and attenuates TNFα gene expression.

In addition to direct stimulation of various cell types involved in the fight against cancer, β-glucan was also found to ameliorate the negative side effects of chemotherapy, including immunosuppression. Recent observation demonstrated that β-glucan-based alleviation of cyclophosphamide-induced severe immunosuppression is caused by regulation of gut microbiota [113].

Another possible mechanism is the inhibition of transformation induced by oncogenes. This was demonstrated with glucans from *Ganoderma lucidum* and *Tricholoma lobayence* using cell transformation caused by *ras* oncogene. Both glucans successfully inhibited cell transformation [114]. The fact that this inhibitory effect required the presence of normal cells cannot be presently explained.

Numerous molecular targets of mushroom-derived β-glucan involve NF-κB inhibitors, protein kinase inhibitors, modulators of G1/S and G2/M checkpoints, inhibitors of MAPK protein kinase signaling pathways, cyclooxygenase inhibitors, and DNA polymerase inhibitors (for review, see Zaidman, Yassin, Mahajna, and Wasser (2005) [93]). In cases of lentinan, recent observation suggested that the breast cancer progression inhibition is modulated via the Nur77/HIF-1α axis [115]. The authors speculate that HIFs might be a potential target in breast cancer treatment.

Some mushroom β-glucans can have positive effects on cancer development manifested indirectly. Inflammatory bowel diseases have a clear connection with the development of colorectal cancer; colitis-associated colorectal cancers form over 5% of all colorectal cancers [116], with some studies reporting as high as 43% [117]. β-glucan administration does not only attenuate inflammation and other symptoms of colitis [118], but prevents carcinogenesis via inhibition of P450 1A2 expression [119]. Similarly, oral supplementation with *Pleurotus*-derived β-glucan suppressed expression of proliferation-associated marker proliferating cell nuclear antigen, meaning that β-glucan administration will suppress abnormal proliferative activity of pre- and neoplastic cells, and subsequently suppress the development of cancer [120].

An unknown mechanism was found in a study of β-glucan from *Sparassis crispa*. Using colon cancer as a model, this β-glucan exhibited direct toxicity against various human colon cancer cell lines by destroying membrane integrity, whereas normal colon cells were resistant [121]. β-glucan direct toxicity is extremely rare, so currently there is no explanation of these effects.

Even though most of the effects of β-glucan are manifested via binding to some of the numerous specific receptors, few studies describing direct effects of β-glucan on cells expressing none of these receptors exist. A glucose-based polysaccharide isolated from *Ganoderma lucidum* was found to have direct effects on lung cancer cells, probably via inhibition of phosphorylation of several signaling molecules [122]. A β-glucan from *Pleurotus djamor* used in high doses inhibited and killed ovarian carcinoma cells PA1 [123]. Surprisingly, soluble β-glucan isolated from the same mushroom had no direct cytotoxic effects. Proteoglucan from *Grifola frondosa* modulated expression of some cancer-related genes in both canine and human tumor cells [124]. Our laboratory found somewhat similar results using synthetic glucan-based oligosaccharides [125]. Some direct effects on lung cancer cells were also described for β-glucan from *Antrodia cinnamomea,* with the supposed mechanism being regulation of the TGFβ/AKT/GSK3β axis [126]. β-glucan from *Lentinus edodes* can in some cases inhibit proliferation of breast cancer cells when used simultaneously with hypoxic conditions [115]. The problem of all these studies, however, is the fact that none of them were ever independently repeated. In addition, they all differ in type of β-glucan and in experimental conditions. Therefore, the possible direct effects of β-glucan remain a side note.

## 4. The Antitumor Effect of β-Glucan in Humans

Mushroom-derived β-glucan-rich polysaccharides are known for their immunomodulatory and antitumor properties. The polysaccharide fraction, mainly β-glucans, is responsible for the immunomodulatory effects. Fungal β-glucans have been proven to activate leukocytes, and this action depends on structural characteristics of β-glucans [127]. Additionally, these compounds may be employed as drug carriers. β-Glucan research is now focused on human studies: clinical trials and epidemiological assessment of the efficacy and safety of mushroom-derived β-glucans for cancer treatment and prevention [128]. Fungal β-glucans can be used as adjuvants for treating cancer patients [129,130]. In general, β-glucans are a promising option for cancer prevention and treatment, especially for cervical cancer. The therapeutic potential of β-glucan alone or as an adjuvant therapy in cervical cancer has been documented; moreover, some authors highlighted β-glucans as drug carriers for preventive and therapeutic use [131]. Glucans and specific proteins are responsible for most of the biological effects of mushrooms, particularly in terms of immunomodulatory and antitumor activities [129]. Human studies represent a minority of the available data, as exemplified by placebo-controlled trials.

β-1,3-d-Glucans and β-1,6-d-glucans (polysaccharides from higher fungi) increase the number of Th1 lymphocytes, which help to prevent allergic reactions. Some β-glucans, like pleuran from oyster mushrooms (*Pleurotus* spp.) or lentinan from shiitake mushrooms (*Lentinula edodes*), have a marked anticarcinogenic activity. In addition to having an immunostimulatory effect, β-glucans may participate in the physiological processes related to the metabolism of fats in the human body. Their therapeutic application causes a decrease in the total cholesterol content of the blood and may contribute to reductions in body weight [132].

Vetvicka and Vetvickova [17,36] compared five different β-glucans, isolated from algae, yeast, bacteria, oats, and a mushroom, by studying their promotion of the phagocytosis of blood cells and the secretion of IL-2, and their suppression of melanoma and breast and lung cancers. In addition, those authors evaluated the impact of β-glucan supplementation on two experimental models of infection. It was concluded that most of the tested β-glucans stimulated phagocytosis and IL-2 secretion, reduced cancer growth, and ameliorated the effects of the experimental infections. Some clinical trials of fungal β-glucans as an adjuvant therapy for cancer started to appear in the literature in the 1980s [130].

Dietary β-glucans are soluble fiber with possible health-promoting effects. Gut peptides serve as important signals for the regulation of energy and glucose homeostasis. This article reviews the effects of different foods enriched in β-glucan on immune responses, inflammation, gut hormones, and cancer. Gut hormones are influenced by the consumption of β-glucan-enriched foods, and in humans, the levels of such peptides as ghrelin and glucagonlike peptides 1 and 2 influence serum glucose concentration, as well as innate and adaptive immunity. Cancer cell progression is also regulated by obesity and glucose dyshomeostasis, which are affected by β-glucan consumption with food, in turn regulating gut hormones [46].

β-glucan alters inflammation via immunostimulatory patterns [133,134]. This phenomenon may be related to a possible effect on gut hormone control (Huang, 2015), although there are opposite opinions [2].

Macrophages perform an important function in all phases of host defense in innate and adaptive immunity through the secretion of cytokines (IL-1, IL-6, IL-8, IL-12, and TNF-α) and inflammatory mediators like nitric oxide (NO) and hydrogen peroxide (H_2_O_2_) [46]. Moreover, as reported recently, β-glucan (a Dectin-1 ligand) promotes macrophage M1 polarization via the NF-κB/autophagy pathway. Additionally, Dectin-1 small interfering RNA (siRNA), autophagy inducer rapamycin, and NF-κB inhibitor SN50 reverse the impact of β-glucan on the autophagy level and macrophage M1 polarization, suggesting that Dectin-1 and NF-κB act upstream of autophagy [135].

According to the data of Wang, Wu, Chen, Liu, and Chen (2015) [6], obtained in an experimental Lewis lung carcinoma tumor model, oral treatment with *Ganoderma lucidum* or *Antrodia camphorata* polysaccharides significantly reduced the TGFβ release into blood serum. Simultaneously, it was shown that oral mushroom β-glucan treatment significantly increased IFNγ mRNA expression but significantly reduced COX2 mRNA expression in the lungs. In addition, IL-12 and IFNγ mRNA expression was significantly increased, but IL-6, IL-10, COX2, and TGFβ mRNA expression levels were substantially lowered following oral treatment with mushroom polysaccharides. This highlights a strong beneficial effect of mushroom polysaccharides against immune suppression in the tumor microenvironment [104]. An enhanced M1 phenotype of tumor-associated macrophages (TAM) and their attenuated M2 phenotype could be achieved by ingesting mushroom polysaccharides.

Recently, researchers isolated and compared α- and β-glucans from shiitake mushrooms (*L. edodes*) with different biological activities [136,137]. A polysaccharide-enriched extract obtained from *L. edodes* was subjected to several purification steps to separate three d-glucans containing β-(1,6), β-(1,3), α-(1,6), and α-(1,3) linkages, with subsequent characterization by nuclear magnetic resonance spectroscopy, gas chromatography coupled with mass spectrometry, infrared spectroscopy, size exclusion chromatography, and other methods.

**Figure 5:**
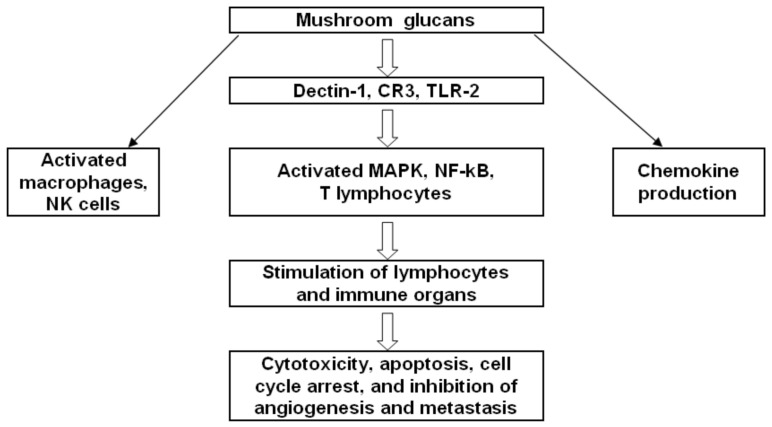
A probable immunomodulatory mechanism of the action of mushroom glucans.

The anticancer effect of β-glucans may be related to their control of inflammation via immunostimulatory patterns [46]; on the other hand, it might be associated with a possible influence on the control of gut hormones [133]. When β-glucans are employed as immunostimulatory agents or adjuvant therapeutics, several receptors have been reported to recognize β-glucans, including Dectin-1, complement receptor 3 (CR3), CD5, and lactosylceramide [138] (Figure 5). Bose et al. [3,4] investigated the participation of various receptors (Dectin-1 and CR3, involved in the oxidative burst) in response to different physical forms of β-glucans in human monocytes.

Furthermore, β-glucans can induce the proliferation of human peripheral blood mononuclear cells and maturation of monocyte-derived dendritic cells via cytokine production [139]. Therefore, it seems that β-glucans can stimulate a broad immune response including phagocytosis and proinflammatory events, which may lead to the elimination of infectious agents (e.g., *Staphylococcus aureus*, *Escherichia coli*, *Candida albicans*, *Pneumocystis carinii*, *Listeria monocytogenes*, *Leishmania donovani*, and an influenza virus). Several studies have confirmed in animal models that systemic treatment with β-glucans enhances the migration of neutrophils into a site of inflammation and improves their antimicrobial function [140].

**Figure 6:**
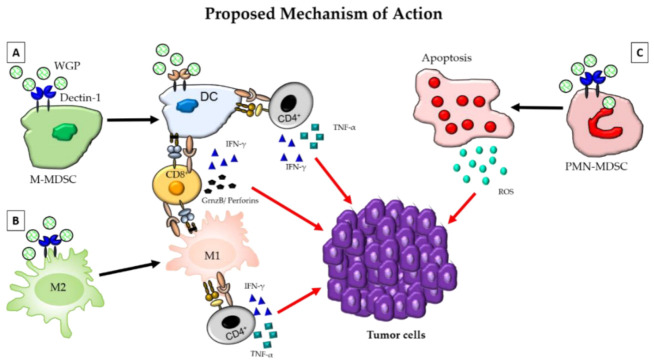
The modulation of the immune cells in the tumor microenvironment by β-glucan. β-glucan binds to the Dectin-1 receptors expressed on cells of the myeloid lineage and is then phagocytosed. In (**A**), β-glucan can be seen binding to Dectin-1 on an M-MDSC. Binding to the M-MDSC will cause the M-MDSC to switch from a suppressive phenotype to a DC phenotype that can act as an APC. This dendritic cell (DC) will then activate CD4^+^ and CD8^+^ T-cells, where CD4^+^ T-cells will secrete pro-inflammatory cytokines, such as TNFα and IFN-γ, and CD8^+^ T-cells will secrete Granzyme B, perforins, and IFN-γ. The secretion of these pro-inflammatory cytokines by CD4^+^ and CD8^+^ T-cells will lead to the destruction of tumor cells. β-Glucan induces the polarization of suppressive M2 macrophages (**B**) into inflammatory M1 macrophages. M1 macrophages will then activate Th1 type T-cells, leading to damage to the tumor cells through the secretion of pro-inflammatory cytokines by CD4^+^ and CD8^+^ T-cells. Finally, in (**C**), β-glucan will bind to the Dectin-1 receptor on polymorphonuclear (PMN)-MDSCs and cause apoptosis of the cell. As the cell undergoes apoptosis, it will produce ROS that will ultimately target the tumor cells, leading to tumor cell death. Overall, these mechanisms together convert a suppressive tumor microenvironment (TME) to an inflammatory TME that has a greater potential to induce the killing of tumors. From Geller, Shrestha, and Yan (2019) [109].

Mushroom compounds modulate the immune system, thereby helping it to fight tumors and other diseases. These compounds can strengthen the immune system by stimulating lymphocytes, NK cells, and macrophages; by enhancing cytokine production; by inhibiting the proliferation of cancer cells; by promoting apoptosis; and by blocking angiogenesis, in addition to being cytotoxic to cancer cells. These compounds encounter intestinal cells, the frontline of the intestinal immune system, and interact with antigens, thereby taking part in an intestinal immune response and inducing an inflammatory response if necessary. Figure 6 shows the modulation of the immune cells in the tumor microenvironment by β-glucan.

Studies of glucans in human cancers include clinical trials and epidemiological data related to the efficacy and safety of mushroom-derived β-glucans in cancer treatment and prevention [128]. Recently, Medicinal Mushrooms Physician Data Query (PDQ): Health Professional Version [141] presented a cancer information summary for health professionals, providing comprehensive, peer-reviewed, evidence-based information about the use of medicinal mushrooms in the treatment of patients with cancer. According to recent research, β-glucans have a variety of potential therapeutic properties as well as metabolic and beneficial effects on the gastrointestinal tract, and hold promise for further clinical application [142]. Therapeutic effects of fungal β-glucans on human colon cancer were documented [143]; the β-glucans decreased the size of xenografted colon cancer tumors via the stimulation of the immune system and direct cytotoxicity. This type of polysaccharide can also exert synergistic effects with chemotherapeutic agents and other drugs (immune stimulators). An innovative strategy is to utilize β-glucans to deliver nanoparticles containing chemotherapeutic agents to the site of colon cancer, thus improving therapeutic efficacy. Lentinan is a component of the second most cultivated and most popular edible mushroom in the world, known as “xianggu” in China and “shiitake” in Japan. There are 9474 reported cases of lentinan-associated cancer treatments, including lung cancer (3469 cases), gastric cancer, and other malignant tumors [94]. A comprehensive review of mushroom β-glucans in cancer therapy suggested stimulation of immune system as the basic mechanism of action [128]. Fungal β-glucans are promising as an adjuvant therapy for cancer [130], as well as modulating the immune system weakened by radiotherapy and chemotherapy in cancer treatment [32].

## 5. Tumor-Associated Macrophages

TAM play important roles in the progression of many malignant solid tumors, including breast cancer [144]. Strategies for the construction of a polysaccharide-based drug delivery system involving three types of β-d-glucan, including mushroom and yeast β-d-glucans as well as a hyperbranched β-d-glucan, have been discussed with reference to their branching characteristics and conformation, as well as their applications as a nano-carrier for the delivery of drugs targeting TAM [145]. TAM are considered M2-like macrophages, but they are much more complex than M2-like macrophages that are typically induced by interleukin (IL)-4/IL-13 signaling. Many signaling pathways in TAM are triggered by factors within TAM [146]. A broad arsenal of molecules is subsequently produced by TAM and executes tumor-promoting functions [147,148]. In a model of Lewis lung carcinoma, a strong effect of mushroom polysaccharides taken orally was noted, namely, amelioration of the immune suppression in the tumor microenvironment [6]. Moreover, those authors presented data indicating that the M1 phenotype of TAM can be enhanced, while the M2 phenotype of TAM can be attenuated by the ingestion of mushroom polysaccharides. According to the results obtained by de Graaff et al. [149], some polysaccharides (yeast-derived β-glucan, lentinan, curdlan, and zymosan) can reshape TAM into producers of inflammatory chemoattractants. Thus, β-glucans and zymosan have the unique ability to preferentially skew macrophages toward a chemoattractant-producing phenotype, which may enhance anticancer immune responses [32].

Transcription factor EB (TFEB) overexpression favorably modulates TAM gene expression through multiple signaling pathways. For instance, TFEB upregulates suppressor of cytokine signaling 3 (SOCS3), peroxisome proliferator-activated receptor γ (PPARγ), and autophagy/lysosome activities, and also inhibits NLRP3 (NLR family pyrin domain-containing 3) and an inflammasome-mediated and hypoxia-inducible factor (HIF)-1α–mediated hypoxia response, thereby downregulating an array of effector molecules in TAM, including arginase 1, IL-10, IL-1β, IL-6, and prostaglandin E2. It was concluded that TFEB is an important regulator of TAM in breast cancer; it controls TAM gene expression and function through multiple autophagy/lysosome-dependent and independent pathways. Therefore, pharmacological activation of TFEB may be a promising approach to improving the efficacy of existing treatments, including immunotherapies of breast cancer because of favorable modulation of TAM function and of the tumor microenvironment [144].

## 6. Conclusions

Thousands of studies clearly demonstrating bioactive effects of β-glucans together with numerous clinical trials strongly suggest that β-glucan has great potential to become a drug used in the treatment or prophylaxis of various diseases. However, the conclusions and full revelation of mechanisms of action are hampered by differences in sources, doses, isolation, and physicochemical properties of the glucans studied [7]. Lately, there is growing interest in investigating the effects of mushrooms on immunity (including autoimmunity) and cancer, and into bioactive properties of the main active ingredients of mushrooms—β-glucans—and molecular mechanisms of their action. The molecular basis for the antitumor properties of a mushroom polysaccharide used as a cancer drug in China was investigated more thoroughly [150]. At the same time, there is an increasing number of research articles about the properties of β-glucans isolated from different sources (e.g., mushrooms). It has been demonstrated that β-glucans are responsible for most of the biological effects of mushrooms, especially for immunomodulatory and antitumor actions. To date, numerous studies have been performed on cell lines and murine models, but there are fewer clinical trials in this field. Several mushroom-derived β-glucans, such as lentinan, possess a marked anticarcinogenic activity along with an immunostimulatory effect. Additionally, β-glucans may affect physiological processes related to lipid metabolism in the human body. Further studies on the possible antimetastatic effects of various β-glucans would be worthwhile.

β-Glucans from various sources seem to have chemoprotective effects according to experimental models and clinical studies. Furthermore, it is important to investigate the actions of β-glucans from different sources on immune cells having antitumor functions, which might cause tumor regression and can influence innate and adaptive immunity. Only a few clinical trials have been conducted regarding the efficacy of purified β-glucans in combination with anticancer drugs. Therefore, additional well-designed clinical trials are needed to verify the reported clinical efficacy of β-glucans. Long-term studies should be undertaken to determine whether, and to what extent, gut peptide levels are affected by foods enriched in β-glucans. This information may be helpful for characterizing the possible health benefits of long-term consumption of β-glucan-enriched foods.

## Figures and Tables

**Table 1:** Mushroom-derived β-glucans.

Name	Source	Type of Polymer
Lentinan	*Lentinus edodes*	Linear
Pleuran	*Pleurotus ostreatus*	Branched
Pachymaran	*Poria cocos*	Linear
Schizophyllan	*Schizophyllum commune*	Branched
Grifolan	*Grifola frondosa*	Branched
Maitake	*Grifola frondosa*	Branched
Pestolan	*Pestolatia sp.*	Linear
Coriolan	*Coriolus versicolor*	Linear
β-Glucan	*Cryptoporus colvatus*	Branched
Polycan	*Aureobasidium pullans*	Branched
β-Glucan	*Ganoderma lucidum*	Linear
β-Glucan	*Agaricus blazei*	Branched

## Data Availability

Not applicable.
